# Impact of anastomotic leakage and radiotherapy on long-term quality of life after sphincter-saving rectal resections

**DOI:** 10.1007/s00423-025-03799-1

**Published:** 2025-07-15

**Authors:** Rasim Khalilov, Steffen Seyfried, Christoph Reissfelder, Julia Hardt, Florian Herrle, Vugar Yagublu

**Affiliations:** 1 Department of Surgery, Medical Faculty Mannheim, Universitätsmedizin Mannheim, Heidelberg University, Mannheim, 68167 Germany; 2 Department of Surgery, RoMed Clinic, Rosenheim, Germany

**Keywords:** Rectal cancer, TME, LARS, Anastomotic leakage, Quality of life

## Abstract

**Background:**

The impact of anastomotic leakage (AL) on quality of life (QoL) following low anterior resections (LAR) remains a pressing concern, particularly as advancements in surgical techniques and multimodal treatments have resulted in an increasing number of survivors.

**Methods:**

This study evaluated how AL affected health-related QoL in patients treated at the Department of Surgery, University hospital Mannheim of Heidelberg University from 2010 to 2021, utilizing the LARS score, the EORTC QLQ-C30, and the EORTC QLQ-CR29 questionnaires.

**Results:**

The study included 20 patients in each group, with those having AL matched 1:1 to control subjects without AL, based on criteria such as age, gender, comorbidities, tumor location, and resection degree. Both groups showed impaired QoL in EORTC assessments, with no statistically significant differences except in the abdominal pain scale of EORTC QLQ-CR29, which was higher for AL patients (19.99 ± 22.68 vs. 6.66 ± 17.43; *p* = 0.03). Comparison of QoL between patients who received neoadjuvant radiotherapy and those who did not, independent of AL, revealed significantly reduced QoL in several scales of both the EORTC QLQ-C30 and QLQ-CR29 assessments. A statistically significant worsening of QoL was observed in the sore skin domain (36.66±34.81 for RT vs. 3.70±11.11 without RT; *p* = 0.02) of the EORTC QLQ-CR29 among AL patients who received neoadjuvant radiotherapy. AL patients treated with Endoscopic Vacuum Therapy (EVT) showed improved QoL.

**Conclusions:**

AL alone does not appear to be an independent risk factor for impaired QoL after LAR for rectal cancer. However, the combined effect of AL and neoadjuvant radiotherapy may contribute to worse functional outcomes, significantly impacting QoL. Effective AL management with EVT can help mitigate these effects and improve patient outcomes.

## Introduction

The improved survival rates of rectal cancer patients are due to early detection, improved surgical techniques such as total mesorectal excision (TME) and advanced coordination and planning of chemoradiotherapy [1, 2]. These achievements have contributed significantly to the decreased mortality rate of rectal cancer in various countries, especially in Western countries [3, 4]. However, research highlights the ongoing health problems faced by rectal cancer survivors. These include reduced general health and physical well-being, impaired bowel functionality, increased pain, increased financial worries and reduced ability to work [5, 6]. Numerous studies show that these problems are more severe in patients with postoperative complications, such as anastomotic leakage (AL), emphasizing the need for improved long-term management of functional outcomes [7–9].

Sphincter-preserving procedures has become more widely used, particularly with the development of techniques that reduce the distal resection margin required and recent advances in intersphincteric resection [10]. However, this technique is associated with a range of adverse effects, the most common of which is AL [11]. AL following sphincter-sparing rectal resections for rectal cancer has been shown to have an impact on increased risk of local recurrence [12], reduced long-term survival [13, 14], and prolonged hospital stays [15], which have been extensively studied in significant cohorts of patients.

A recent meta-analysis by Snijders et al. involving 22 studies with 10.343 patients showed that AL contributes significantly to overall postoperative mortality and is responsible for one third of all postoperative deaths [16]. Another meta-analysis, which included 21.883 patients from 28 studies and investigated the association between AL and local recurrence after rectal resection, found an increased local recurrence rate in patients with AL with an OR of 1.69 (95% CI, 1.45-1.96; *P* < 0.00001) [12]. Although the number of survivors after rectal resection is increasing, there remains a research gap targeting the long-term quality of life (QoL) of these individuals, particularly with regard to bowel functionality, sexual impairment, psychological problems and social adjustment. This highlights the importance of focusing not only on the primary treatment aspects, but also on the long-term well-being and QoL of cancer survivors, especially those who have undergone complicated therapy. The aim of the study was therefore to investigate the long-term QoL of stoma-free patients with rectal cancer and AL after sphincter-preserving rectal resections who were treated at the Surgical Clinic of the University Hospital Mannheim of Heidelberg University between 2010 and 2021.

## Materials and methods

### Patient characteristics

We conducted a case-controlled retrospective analysis combined with a prospective QoL assessment of patients who underwent rectal resection with primary anastomosis and total mesorectal excision (TME) for mid and lower rectal cancers at our institution between 2010 and 2021 and developed AL. In this case-controlled study, patients with AL were compared with control subjects without AL in a 1:1 ratio, taking into account age, gender, comorbidities, distance of tumor from anal verge and neoadjuvant radiotherapy. Only patients who had undergone a stoma reversal and no longer had a stoma were included in the study. Patients with metastatic disease, acute bowel obstruction, synchronous colorectal carcinoma, T1 rectal carcinoma treated with local excision by transanal endoscopic microsurgery and a history of rectal surgery were excluded from the analysis. Patient characteristics were obtained from the database, including age, gender, comorbidities assessed by the Charlson Comorbidity Index (CCI), distance of tumor from the anal verge, extent of transmural tumor invasion, pathologic N stage, status of neoadjuvant therapy, type of anastomosis, severity of AL and preoperative serum albumin level.

### Location of the tumor

Information on the location of the tumor, which was identified by rigid proctoscopy and documented accordingly, was taken from the patient records and classified as follows: lower rectum (distal tumor margin less than 6 cm from the anal verge), mid-rectum (6–12 cm from the anal verge) and upper rectum (12–16 cm from the anal verge).

### Functional outcome and quality of life assessment

The functional outcomes were assessed using a LARS score validated for German language [17] and two questionnaires from the European Organization for Research and Treatment of Quality of Life Questionnaire for Colorectal Cancer - EORTC QLQ-C30 [18] and EORTC QLQ-CR29 [19]. The LARS scoring system has become widely accepted due to its clear problem definition and strong correlation with QoL [20]. This system comprises five questions that assess the following items: Incontinence with flatus, incontinence with liquid stool, frequency of bowel movements, frequency of bowel movements and urgency [17]. The LARS score ranges from 0 to 42, with a higher score indicating a higher level of symptoms and impaired QoL. As originally described [9], the LARS score was divided into three categories: no LARS (symptoms have no impact on the patient’s quality of life), minor LARS (symptoms have a minor impact on the patient’s QoL) and major LARS (symptoms have a major impact on the patient’s QoL). A score from 30 to 42 corresponds to major LARS, 21–29 to minor LARS, while values 0–20 indicates no LARS.

The EORTC QLQ-C30, on the other hand, comprises five functional domains (physical, functional, emotional, cognitive and social functioning), a global health score and nine cancer-related symptoms [18]. The EORTC QLQ-CR29 includes colorectal cancer-specific domains and symptoms, including items on sexual function, stoma and bowel function [21]. A total of 15 scales of the EORTC QLQ-C30 questionnaire and 23 of the 28 scales of the EORTC QLQ-CR29 questionnaire (without questions on the stoma) were evaluated. The answers to the questions in both questionnaires were recorded on a four -point(from “not at all” to “very much”) and on a seven-point (from “very poor” to “excellent”) Likert scale and converted into a score between 0 and 100, with higher scores representing better functioning on the functional scales and a higher level of symptoms on the symptom scales. A 10-point shift in scores was considered the threshold for a clinically relevant difference [22]. A difference between 10 and 20 points was categorized as moderate, while a difference of more than 20 points was considered severe impairment of patient’s QoL [23]. QoL assessments were conducted in the years 2023 and 2024. All eligible patients received the questionnaires by post, together with a letter explaining the purpose of the study, assuring confidentiality, inviting participation and requesting informed consent.

### Definition and determining the severity of anastomotic leakage

According to the International Study Group of Rectal Cancer (ISREC), AL is defined as a defect in the intestinal wall at the anastomotic site (including suture and staple lines of neorectal reservoirs) that results in a connection between the intra- and extraluminal compartments [24]. The severity of AL was assessed according to the criteria proposed by ISREC and categorized into grades A, B and C. A grade A AL requires no change in patient treatment, while a grade B leak requires active therapeutic intervention but can be managed without repeat laparotomy. In contrast, grade C AL requires a repeat laparotomy.

### Ethical consideration

Our study was conducted after approval of the Medical Ethics Commission II of the Medical Faculty Mannheim, Heidelberg University, Mannheim, Germany (TEMP803797-AF 5). Written informed consent was obtained from all patients included in this study. All investigations were performed according to the Declaration of Helsinki.

### Statistical analysis

JMP 16 (SAS Institute Inc., Cary, NC, USA) was used for the statistical calculations, with significance set at a two-sided p-value of < 0.05. The questionnaires were assessed according to the respective manuals. Scores on the EORTC QLQ-C30 and QLQ-CR29 ranged from 0 to 100, with higher scores expressing better functioning or better general health or with higher levels of symptoms. The mean values for each question of the EORTC QLQ-C30 and QLQ-CR29 questionnaires and the mean LARS score were compared with Wilcoxon-Mann-Whitney U tests in subgroups based on the presence of AL and neoadjuvant radiotherapy. Standard deviations are presented as ± values in parentheses throughout the text.

## Results

During the study period, a total of 1.038 patients underwent sphincter-preserving rectal resections with curative intent. Of these, 575 patients underwent low anterior resection, with 416 of them having rectal carcinomas resected at a distance of up to 12 cm from the anal verge. AL of different severity occurred in 68 patients (6.5%of all rectal resections and 11.8% of LAR). The stoma could be reversed in 55 patients with AL, who were the focus of our study. A total of 23 of these patients passed away during the study period. In all, 20 patients with AL responded to the questionnaires we sent out, and 20 patients without AL were matched as described in the methods section.

Characteristics of the patients, including gender, age, distance of the tumor from the anal verge, extent of transmural tumor invasion, status of neoadjuvant therapy, pathological N-Stage, type of anastomosis, severity of AL, and level of serum albumin are listed in Table 1. There was no statistical difference between the two groups in terms of patient age, comorbidity index, and location of the tumor from the anal verge. It should be noted that the tumor staging reported in the Table 1 refers to the postoperative pathological stage. In three patients who were classified as Stage I postoperatively, preoperative radiochemotherapy had been administered. These patients demonstrated a marked tumor regression, with minimal to no residual tumor tissue observed in the surgical specimens. Accordingly, they were assigned Dworak regression grade 4, indicating a near-complete pathological response.

**Table 1:** Patient characteristics

Characteristics	AL	No AL	*P* value
Sex			
Male	15 (75%)	15 (75%)	-
Female	5 (25%)	5 (25%)	-
Age at operation, years	56.3±8.6	57.4±8.9	0.63
Current age, years	63.2±7,8	65.9±9.5	0.23
CCI index	4±1.6	3.6±1.0	0.41
Distance from anal verge, cm	7.6±3.2	7.8±3.2	0.96
Intersphincteric dissection			
Yes	5 (25%)	5 (25%)	-
No	15 (75%)	15 (75%)	-
Transmural Invasion, %			
T1	3 (15%)	7 (35%)	-
T2	6 (30%)	8 (40%)	-
T3	11 (55%)	5 (25%)	-
T4	-	-	-
UICC Stages			
I	8 (40%)	12 (60%)	-
II	9 (45%)	6 (30%)	-
III	3 (15%)	2 (10%)	-
IV	-	-	-
Pathological *N* stage			
N-	17 (85%)	18 (90%)	-
N+	3 (15%)	2 (10%)	-
Neoadjuvant therapy			
RT	1 (5%)	-	
RCT	10 (50%)	11 (55%)	-
CT	1 (5%)	1 (5%)	-
NNT	8 (40%)	8 (40%)	-
Anastomosis type			-
End-to-side	19 (95%)	18 (90%)	-
End-to-end	1 (5%)	2 (10%)	-
Stapled	15 (75%)	15 (75%)	-
Handsewn	5 (25%)	5 (25%)	-
Severity of anastomotic			
leakage	3 (15%)	-	-
A	14 (70%)	-	-
B	3 (15%)	-	-
C	37.5±5.5	39.3±2.8	0.17
Serum albumin preoperative			

*AL* Anastomotic Leak, *CCI* Charlson Comorbidity Index, *UICC* Union for International Cancer Control, *RT* Radiotherapy, *RCT* Radiochemotherapy, *CT* Only Chemotherapy, *NNT* No Neoadjuvant Therapy

Neoadjuvant radiotherapy was administered in accordance with established clinical guidelines. Neoadjuvant radiotherapy, with or without concomitant chemotherapy, was primarily indicated for patients with tumors located in the middle or lower rectum and for those with more advanced clinical stages, such as uT2N+, uT3, or uT4. In patients included in the study, the rectal resection was performed laparoscopically16(80%), conventional 2(10%)and da vinci robotic system 2(10%) in AL Group and laparoscopically16(80%), conventional 2(10%) an conversion 1(5%) and da vinci robotic system 1(5%) in control Group, with the construction of a side-to-end or end-to-end anastomosis using circular stapler or handsewn. In all patients a protective ileostomy was constructed. Only three patients in no AL group underwent hand-sewn coloanal anastomosis. The tumor distance from the anal verge was 7.6 ± 3.2 cm in patients with AL and 7.7 ± 3.2 cm in those without AL (*p* = 0.96).

Stoma reversal was performed at an average interval of 7.9 ± 3.4 months in patients with AL, compared to 4.6 ± 2.6 months in patients with no AL. The mean follow-up time after stoma reversal was 6.8 ± 3.3 years (range: 1.8–12.8 years) for patients with AL and 8.09 ± 3.1 (range:2,7-14.1 years) for those in the control group. AL was diagnosed in eight asymptomatic patients based on raised inflammatory markers and subsequent endoscopy. In one of these patients, air leakage was observed from intraoperatively placed pelvic drainage. In 12 patients, AL was diagnosed on the basis of clinical symptoms such as abdominal pain, fever and fatigue, complemented by laboratory findings and subsequent endoscopic examinations. Additionally, three of these patients had turbid secretions from the intraoperatively placed pelvic drainage. Two patients were treated with an endoscopically placed transanal drainage tube. Three patients had a CT-guided drainage for a pararectal abscess. EVT was provided for nine patients. Each treatment required from 4 to 10 sessions. Endoscopic assessment of the anastomosis was a standard method of diagnosis in all patients. Endoscopic examination also provided additional details for determining therapeutic strategies. In 11 patients, antibiotic therapy was administered either as standalone treatment or in combination with one of the above mentioned therapies.

The severity of LARS in AL patients did not correlate with the severity of the disease. In patients with no or minor LARS, eight out of ten (80%) were in grade B and two (20%) in grade C. In patients with major LARS, six out of 10 (60%) were in grade B, three (30%) in grade A, and one (10%) in grade C. No association was found between the duration and number of EVT sessions and LARS severity. In patients with no or minor LARS, the number of EVT sessions were 6±1.4 times, while in patients with major LARS, it was 7.2±2.2 times (*p* = 0.4).

The comparison of QoL based on LARS scores showed no statistically significant difference between the two groups. Patients with AL had a mean LARS score of 26.3 ± 10.1 compared to 26,6 ± 10.5 in the control group (*p* = 0.89). Major LARS was recorded in 10 patients (50%) in both groups, with a score of 35.2 ± 3.2 for AL patients and 35.4 ± 3.7 in the control group. Minor LARS was identified in three patients (15%) from AL group and two patients (10%) from control group, with a score of 24.7 ± 4.1 in the patients with AL and 29 ± 0 in the control patients. LARS scores of 14.8 ± 3.1 and 14.4 ± 1.2 were found in seven patients (35%) in AL group and in eight patients in control group, which are in the range of “no LARS” range (0–20).

EORTC QLQ-C30 assessment demonstrated (Table 2) a comparable levels of change in the global health status of patients in both the AL and control groups (69.58 ± 20.46 vs. 71.24 ± 18.43; *p* = 0.70).The differences in changes across various QoL scales of EORTC QLQ-C30 were largely statistically non-significant and not clinically relevant between the two groups. The clinically relevant changes was detected only in the domains of dyspnea (23.33 ± 28.81 vs. 10.83 ± 15.55; *p* = 0.18) and diarrhea (23.33 ± 26.71 vs. 44.99 ± 36.31; *p* = 0.05). It is well accepted that even though the EORTC results are not statistically significant, a difference of 10 or more points on the 0–100 scale of the EORTC QLQ-CR29 and EORTC QLQ-C30 scores between the two comparison groups is considered clinically relevant change and an indication of a significant impact on a patient’s QoL [22].

**Table 2:** EORTC QLQ-C30 scores of AL and no AL groups

Variable	AL (Mean)	No AL (Mean)	*p*-value
Global health status	69.58	71.24	0.70
Physical functioning	81.99	87.33	0.38
Role functioning	71.67	76.66	0.61
Emotional functioning	75.41	72.50	0.81
Cognitive functioning	87.49	86.66	0.69
Social functioning	65.83	68.33	0.69
Fatigue	28.33	24.99	0.69
Nausea and vomiting	5.00	1,67	0.21
Pain	20.83	19.72	0.77
Dyspnea	23.33	10.83	0.18
Insomnia	33.33	33.33	0.97
Appetite loss	6.66	6.66	0.76
Constipation	13.33	15.00	0.93
Diarrhea	23.33	44.99	0.05
Financial difficulties	13.33	13.33	0.29

*AL* Anastomotic Leak No, *AL* No Anastomotic Leak.

The EORTC QLQ-CR29 (Table 3) showed statistically significant changes in the QoL scales of altered body image and abdominal pain in patients with AL compared to patients without AL (altered body image: 70.75 ± 22.25 vs. 82.77 ± 24.04; *p* = 0.04, abdominal pain: 19.99 ± 22.68 vs. 6.66 ± 17.43; *p* = 0.03). A clinically relevant worsening was documented in the domain of stool frequency (36.66 ± 30.87 vs. 49.99 ± 29.11; *p* = 0.17) in the patients without AL. In all other QoL scales, the median scores demonstrated no statistically or clinically significant differences.

**Table 3:** EORTC QLQ-CR29 scores of AL and no AL groups

Variable	AL (Mean)	No AL (Mean)	*p*-Value
Body image	70.00	82.77	0.04
Anxiety	55.00	68.33	0.15
Weight	75.00	76.66	0.98
Sexual interest (men)	46.66	55.55	0.53
Sexual interest (women)	53.33	33.33	0.36
Urinary frequency	44.16	40.25	0.65
Blood and mucus in stool	7.50	7.10	0.72
Stool frequency	36.66	49.99	0.17
Urinary incontinence	16.66	25.00	0.44
Dysuria	0	3.33	0.16
Abdominal pain	20.00	6.66	0.03
Buttock pain	18.33	19.99	0.71
Bloating	33.33	28.33	0.68
Dry mouth	11.66	16.66	0.60
Hair loss	8.33	1.66	0.16
Taste	10.00	3.33	0.35
Flatulence	38.33	43.33	0.59
Fecal incontinence	21.66	31.66	0.37
Sore skin	21.66	33.33	0.22
Embarrassment	20.00	29.99	0.38
Impotence	55.55	60.00	0.68
Dyspareunia	19.99	26.66	0.91

*AL* Anastomotic Leak No, *AL* No Anastomotic Leak.

We conducted a comparative analysis of QoL between patients who received neoadjuvant radiotherapy prior to surgery and those who did not, independent of AL. In this comparison, the median LARS score was higher in patients who received neoadjuvant radiotherapy (28.68±0.10.60; 22 patients vs. 23.72±9.05; 18 patients), but this difference did not reach statistical significance (*p* = 0.13). Consistent trends of reduced QoL associated with neoadjuvant radiotherapy were observed in the results of the EORTC QLQ-C30 (Table 4) and EORTC QLQ-CR29 (Table 5) results.

**Table 4:** EORTC QLQ-C30 scores of RT (neoadjuvant radiotherapy) and no RT groups (no neoadjuvant radiotherapy)

Variable	RT (Mean)	No RT (Mean)	*p*-Value
Global health status	68.56	72.68	0.52
Physical functioning	85.15	84.07	0.35
Role functioning	76.51	71.30	0.36
Emotional functioning	71.97	76.38	0.84
Cognitive functioning	84.84	89.81	0.69
Social functioning	65.90	68.51	0.89
Fatigue	28.28	24.70	0.92
Nausea and vomiting	3.78	2.77	0.86
Pain	15.15	26.54	0.25
Dyspnea	21.21	12.03	0.24
Insomnia	31.81	35.18	0.84
Appetite loss	4.54	9.25	0.45
Constipation	9.09	20.37	0.20
Diarrhea	33.33	35.18	0.92
Financial difficulties	13.63	12.96	0.82

*RT* Neoadjuvant Radiotherapy No, *RT* No Neoadjuvant Radiotherapy.

**Table 5:** EORTC QLQ-CR29 scores of RT (neoadjuvant radiotherapy) and no RT groups (no neoadjuvant radiotherapy)

Variable		RT (Mean)	No RT (Mean)	*p*-Value
Body image	70.70		83.33	0.17
Anxiety	65.15		57.40	0.34
Weight	78.78		72.22	0.50
Sexual interest (men)	48.14		55.55	0.55
Sexual interest (women)	41.66		44.44	1.0
Urinary frequency	41.13		43.51	0.69
Blood and mucus in stool	7.57		7.03	0.50
Stool frequency	43.93		42.59	0.80
Urinary incontinence	21.21		20.37	0.96
Dysuria	3.03		0.00	0.21
Abdominal pain	12.12		14.81	0.83
Buttock pain	21.21		16.66	0.52
Bloating	27.27		35.18	0.61
Dry mouth	16.66		11.11	0.51
Hair loss	0.00		11.11	0.01
Taste	6.06		7.40	0.81
Flatulence	45.45		35.18	0.43
Fecal incontinence	34.84		16.66	0.04
Sore skin	36.36		16,66	0.05
Embarrassment	30.30		18.51	0.25
Impotence	61.11		52.77	0.56
Dyspareunia	41.66		11.11	0.19

*RT* Neoadjuvant Radiotherapy No,*RT* No Neoadjuvant Radiotherapy.

A statistically significant worsening of QoL was observed in the scales of EORTC QLQ-CR29 for fecal incontinence (34.84±29.95 for RT vs. 16.66±20.60 without RT; *p* = 0.04) and sore skin (36.36±33.97 for RT vs. 16.66±26.20 without RT; *p* = 0.05) among patients who received neoadjuvant radiotherapy. In addition, the RT group had higher scores for flatulence (45.45±36.43 vs. 35.18±29.10; *p* = 0.43) and embarrassment due to bowel movement (30.30±33.97 vs. 18.51±28.50; *p* = 0.25), while no-RT group had a higher body image score (70.70±26.90 vs. 83.33±17.56; *p* = 0.17) with mean differences between the groups of more than 10 points, suggesting clinical relevance.

The comparative analysis within the AL group, between patients who received neoadjuvant radiotherapy prior to surgery and those who did not, shows that the median LARS score was higher in patients who received neoadjuvant radiotherapy (27.90±11.17; 11 patients) compared to patients without neoadjuvant radiotherapy (24.44±8.74; 9 patients), though this difference was not statistical significant (*p* = 0.38). A statistically significant worsening of QoL was observed only in the sore skin scale of the EORTC QLQ-CR29, with scores of 36.66±34,81 for the RT group versus 3.70±11.11 for the no RT group (*p* = 0.02) among patients who received neoadjuvant radiotherapy. Additionally, comparison of other EORTC QLQ-CR29 scales between the RT and no RT groups demonstrated clinically relevant negative effects of radiotherapy in scales for fecal incontinence (27.27±25.02 vs. 14.81±17.46; *p* = 0.28) and embarrassment because of bowel movement (24.24±30.15 vs. 14.81±24.21; *p* = 0.51), with mean score difference between groups greater than 10. Interestingly, after excluding patients who did not undergo radiotherapy, the comparison of QoL between those with and without AL revealed no statistically or clinically significant differences in the aforementioned scales, specifically fecal incontinence (14.81±17.56 vs. 18.50±24.21; *p* = 0.88) and embarrassment because of bowel movement (14.80±24.20 vs. 22.22±33.33; *p* = 0.68).

Furthermore, we conducted a comparative analysis within the AL group between the patients who received VAC therapy and those who did not. The results showed that the patients who received VAC therapy had statistically significantly better scores on emotional (87.03 ± 16.02 for VAC therapy vs. 65.90 ± 23.90 without VAC therapy; *p* = 0.03) and social functioning (79.63 ± 20.00 vs. 54.54 ± 29.90; *p* = 0.03) and embarrassment due to bowel movement (3.70 ± 11.10 vs. 33.33 ± 29.80; *p* = 0.02), but not in the scale for diarrhoea (36.36 ± 27.70 vs. 7.40 ± 14.70; *p* = 0.01). In addition, clinically relevant improvements were observed in faecal frequency (22.22 ± 25.00 vs. 48.48 ± 31.14; *p* = 0.06) and faecal incontinence (14.81 ± 17.60 vs. 27.27 ± 25.02; *p* = 0.27) for the patients received VAC therapy.

## Discussion

Despite conflicting results in studies on the impact of AL on long-term quality of life after rectal resection, most confirm that AL is an independent risk factor that negatively affects patients’ QoL. This underlines the need for further studies on the challenges faced by these patients and the development of effective treatment strategies [1, 25–28]. A recent systematic review summarized 13 studies evaluating QoL with a focus on AL after rectal resection [29], comparing 138 AL patients with 227 non-AL patients in six case-controlled studies and 289 AL patients versus 2.514 patients in six non-randomized retrospective studies. The results suggest that AL may have an impact on pelvic dysfunction and QoL, although a limited number of patients were studied and differing scoring systems were used. This underscores the importance of conducting additional studies focusing on AL patients, especially regarding the long-term impact on QoL. Therefore, we analyzed the retrospective data and current quality of life of AL patients after LAR with TME performed in our hospital between 2010 and 2021 to consider the factors affecting QoL and to evaluate the treatment modalities involved.

A careful study of the long-term impact of AL on the QoL of these patients could provide valuable insights for improving the treatment and prevention of AL. The mean follow-up time after stoma closure in our study was 6.8 ± 3.3 years for AL patients and 8.09 ± 3.1 years for control subjects, which provides valuable insight into the long-term problems of these patients. According to our study, the impairment of QoL in patients with AL after TME was not significantly different from that of patients without AL. However, within the AL group, those who received neoadjuvant radiotherapy experienced a clinically significant reduction in QoL compared to those who did not.

In our study, 50% of the patients both the AL and control groups had major LARS, compared to a range of 10–72.1% reported in other prospective cohorts with differing patient characteristics, especially in relations to tumor localization and neoadjuvant radiotherapy [6, 30–34]. We showed that the patients with AL who received neoadjuvant radiotherapy experienced a significantly higher incidence of major LARS (63,6%) than those who did not (33,3%), demonstrating a deterioration in QoL and supporting trends reported in the literature.In a meta-analysis of 36 studies on the incidence of LARS after sphincter-preserving surgery for rectal cancer, Sun et al. found that the average incidence of major LARS was 44%, with a wide range from 10–72.1% [34]. In a comparable Dutch study that included patients with AL, 46% of the patients reported major LARS at a median interval of 14.6 years, with a higher rate in the group with neoadjuvant radiotherapy than in the group without radiotherapy (56% vs. 35%, respectively) [33]. Similarly, in a Danish population-based cross-sectional study of 938 patients, major LARS was documented in 41%, including patients with AL [35]. In the subgroup of irradiated patients, major LARS was detected in 64.4%, whereas among patients after LAR without RT, major LARS was found in 34.8%.

Our study provides further confirmation that radiotherapy has a negative impact on the QoL of patients after (TME) for rectal cancer. We showed that neoadjuvant radiotherapy was associated with a statistically significant worsening of quality of life in fecal incontinence and sore skin among rectal cancer patients, as measured by the EORTC QLQ-CR29. Additionally, clinically relevant worsening was observed in the flatulence and embarrassment due to bowel movements scales in patients who received neoadjuvant radiotherapy. Interestingly, the EORTC QLQ-C30 analysis revealed a significantly higher diarrhoea score in the group without AL. One possible explanation for this finding is that patients who experienced AL had significantly more severe symptoms and underwent more intensive postoperative treatment and follow-up care. This may have influenced their perception and reporting of symptoms, leading them to perceive and describe unformed stools as less distressing. Furthermore, given the relatively small sample size of the study, individual variability and potential recall bias cannot be ruled out as influencing factors.

It is well studied that neoadjuvant radiotherapy carries a high risk of side effects and is associated with impaired postoperative bowel, bladder and sexual function - in particular fecal incontinence and erectile dysfunction, which negatively affects QoL. Therefore, the indication for radiotherapy should be carefully reconsidered. According to the MERCURY and OCUM studies, patients with locally advanced rectal cancer in the middle or lower third of the rectum can be effectively selected for neoadjuvant chemoradiotherapy (nRCT) based on the MRI-derived circumferential resection margin (mrCRM), independent of clinical nodal (cN) status [36, 37]. Therefore, nRCT should not be universally applied to all patients with tumors in the middle or lower third of the rectum at cII and III stages but should be reserved for those with high-risk features. The ongoing SELREC trial also aims to demonstrate that omitting neoadjuvant therapy in selected patients with locally advanced rectal cancer and low risk of recurrence, based on preoperative MRI findings, is not inferior to the standard approach of neoadjuvant radiochemotherapy.

On the other hand, our present study also indicates that the combined impact of AL and neoadjuvant radiotherapy might impair the functionality of the reconstructed anorectal tract and results in a clinically significant impairment in QoL for patients. Various studies have shown that radiotherapy can lead to anorectal dysfunction due to vascular toxicity, fibrosis of the anal sphincter, pudendal nerves and myenteric plexus [38–40]. We hypothesize that AL-related inflammation, infection, structural damage and neuromuscular impairment exacerbate the negative effects of radiotherapy on the neorectum through increasing tissue damage and functional deficits. A deeper understanding on these mechanisms would contribute in improvements of therapeutic approaches and enhance QoL. For instance, a recent study by van der Sande et al. showed promising results in minimizing the negative effects of radiotherapy by reducing the dose while maintaining the oncological effect [41]. It should also be considered that neoadjuvant radiotherapy may decrease the success rate of effective AL treatment modalities, such as EVT [42]. The study by Kühn et al. showed that neoadjuvant radiotherapy significantly increased the risk of EVT treatment failure [43]. In our study, no correlation was found between the duration or frequency of VAC therapy sessions and the severity of LARS. However, QoL assessment revealed that patients who underwent EVT had a statistically significant improvement in QoL compared to those who did not.

It should be noted that various organ preservation strategies are currently being investigated as effective alternatives to total mesorectal excision (TME) in the treatment of rectal cancer, with the goal of improving long-term QoL. Recent multicenter data from the UK and Sweden suggest that short-course radiotherapy (SCRT) combined with contact X-ray brachytherapy (CXB) offers a viable organ preservation strategy in predominantly elderly or comorbid patients with early-stage rectal cancer, achieving high rates of clinical complete response and stoma avoidance, thereby reinforcing the importance of assessing long-term quality of life outcomes in non-operative management pathways [44]. On the other hand, new clinical studies highlight the efficacy of radiotherapy when integrated into total neoadjuvant therapy (TNT), reinforcing its value as a key component in the curative treatment of rectal cancer [45, 46]. Although the OPRA trial did not directly assess patient-reported quality of life outcomes, organ preservation through TNT is plausibly associated with improved long-term bowel function and overall quality of life, primarily due to the avoidance of total mesorectal excision (TME) and its associated morbidities [47]. The trial demonstrated that TNT followed by a watch-and-wait (WW) strategy in patients achieving a complete or near-complete clinical response resulted in sustained organ preservation in approximately 50% of patients with stage II/III rectal cancer. Notably, patients in the chemoradiotherapy followed by consolidation chemotherapy (CRT-CNCT) arm achieved significantly higher TME-free survival compared to those receiving induction chemotherapy followed by chemoradiotherapy (INCT-CRT) (54% vs. 39%, *p* = 0.012). These findings underscore the evolving role of radiotherapy within TNT protocols, not only for local tumor control but also as a means to potentially reduce long-term bowel dysfunction by enabling non-operative management in selected patients.

Another factor that contributes to a major LARS as well as AL, is the distance of the tumor from the anal verge [48]. In our study, there was no statistical difference between tumor height in patients of both study groups. An international randomized controlled trial (MRC/NIHR ROLARR) showed that a decrease in tumor height of 1 cm above the apex of the anal canal (OR = 1.290, 95% CI: 1.101–1.511) was a predictor of major LARS [49]. Intersphincteric dissection has not been identified as an independent factor for QoL impairment in our study, even when stratified by neoadjuvant radiotherapy, considering that the small number of patients (five patients in each group) could influence this outcome.

The studies focusing on assessment of QoL in patients with AL after LAR, which used different scoring systems, showed contradictory results [1, 26, 27]. Regardless of the type of questionnaire used in these studies-such as Wexner scores in Artus et al. [1] and Mongin et al. [46], or the FISI used by Ashburn et al. [26] and Re et al. [27] - the scores for bowel incontinence were higher in the AL group. The study by Ashburn et al. demonstrated [26] that the scores of the Short Form-36 Health Survey (SF-36) were higher in the no AL group in both the physical and mental components. In the study by Marinatou et al. [25], the SF-36 and The Gastrointestinal Quality of Life Index (GIQLI) scores were relatively similar during the three-month follow-up period. However, at the six-month and especially at the one-year follow-up, the scores of those in the AL group were significantly lower for QoL. A similar trend was observed by Mongin et al. [46], suggesting that the effects of AL persist over a longer period of time.

There are also studies that show no association between AL and patients’ QoL [1, 50]. For example, Artus et al. [1] conducted a retrospective study and found that the results of QoL questionnaires were not associated with the presence of AL. However, as acknowledged in the study itself, these results may be due to the heterogeneous patient population and the retrospective nature of the study. The study also included patients with distant metastases, which may lead to a significant bias as postoperative recovery is impaired in these patients. Similar results were also shown in a study by Rullier et al. [51], although this study is more than two decades old. The management of AL has improved considerably in the last ten years, which has led to significantly different data in more recent studies [1]. The occurrence of AL is considered detrimental to overall bowel function as it causes significant scarring leading to a reduction in overall rectal compliance [52].

Our study had some limitations that should be considered when interpreting the outcomes. The retrospective nature of the study limits the ability to establish causality between the variables and might lead to biases in patient selection and data processing. The relatively small sample size limits the generalizability of our results. Furthermore, the study did not include different surgical approaches, such as transanal total mesorectal excision (taTME), TME with or without a protective stoma, or two-stage delayed coloanal anastomosis, which may affect outcomes and QoL.

## Conclusion

In conclusion, our study suggests that AL alone does not appear to be an independent risk factor for impaired long time quality of life in patients after LAR for rectal cancer. The cumulative effect of AL and neoadjuvant radiotherapy seems to be critical for the impairment of the functionality of the reconstructed anorectal tract, leading to a clinically significant deterioration in QoL in our study. On the other hand, well-managed AL therapy with EVT can help to improve QoL. Future research should involve robust prospective multicentric studies with larger patient populations, different treatment modalities and standardized questionnaires to better assess the cumulative effects of AL after LAR.

## Data Availability

No datasets were generated or analysed during the current study.
